# Genome-Wide Genetic Diversity and Population Structure of *Charybdis feriata* (Crustacea, Decapoda, and Portunidae) Along the Southeast Coast of China Inferred from Genotyping-by-Sequencing (GBS) Approach

**DOI:** 10.3390/genes15111421

**Published:** 2024-10-31

**Authors:** Jie He, Jialin Wu, Litao Wan, Wenjun Xu, Tianyan Yang

**Affiliations:** 1 Zhejiang Marine Fisheries Research Institute, Zhoushan 316021, China; he_0902@126.com (J.H.); wujialin665@gmail.com (J.W.); wan_066934@126.com (L.W.); wjxu1971@hotmail.com (W.X.); 2 Fishery College, Zhejiang Ocean University, Zhoushan 316022, China

**Keywords:** *Charybdis feriata*, population genomics, genetic variation, population structure, single-nucleotide polymorphisms

## Abstract

Background/Objectives: The swimming crab *Charybdis feriata* is an important commercial fishery species and a major economic contributor to the southeast coastal fishing communities in China. Under the scenario of resource decline and shortage in the market over recent years, it has become more urgent and necessary to explore the fine-scale population genetic characteristics of *C. feriata*. Methods: In this study, the genotyping-by-sequencing (GBS) method was used to estimate the genome-wide genetic variation in and population differentiation pattern of *C. feriata* collected from four geographical locations (Zhoushan, Quanzhou, Yangjiang, and Qinzhou) along the southeast coast of China. Results: A total of 18,815 high-quality single-nucleotide polymorphisms (SNPs) were identified and the results revealed the existence of high genetic diversity and low genetic divergence among the populations of *C. feriata*. Floating eggs and larvae transported by alongshore currents during the reproductive season might enhance the interpopulation genetic exchange. Principal component analysis (PCA) and a phylogenetic tree showed a high genetic connectivity of *C. feriata* across the southeast coast of China, but *C. feriata* distributed in the Zhoushan Archipelago might possess some genetic distinctiveness and diversification. Conclusions: The results supplemented basic genetic information of *C. feriata* at the genome level and also provided specific knowledge that could lead to the improved spatial management of fishery resources.

## 1. Introduction

*Charybdis feriata* (Linnaeus, 1758), commonly known as the crucifix crab or red crab, is a commercially important portunid crab, widely spread throughout the tropical and subtropical sea areas of the Indo-West Pacific [1]. As a sublittoral species, it mainly inhabits sandy and muddy bottoms as well as rocky coasts with mudstone and coral reefs, with a water depth of 10–60 m [2,3]. In China, it is extensively distributed along the coastal waters of the southern East China Sea and the South China Sea, and has become an important edible marine crab resource in the southeast coast of China for its delicious flavors and high nutritional value [4]. In recent years, the ability to catch *C. feriata* has been unable to meet the market demand with increasing international trade in eastern Asia and because wildlife populations of *C. feriata* are facing dramatic declines due to overfishing. Meanwhile, environmental changes combined with habitat destruction by human activities are also assumed to induce resource fluctuations substantially, which give rise to profound effects on the resource distributions of this species. However, most of the literature regarding *C. feriata* in the past has focused on fishery resources and fishing [5,6,7,8,9], fishery biology [10,11], and reproductive biology [12,13,14]. Only a smaller number of genetic studies have been reported in comparison to the general biology of this species and fishery until now [15,16,17,18].

Fishery management requires not only an understanding of the biological principles underlying resources, but also a deeper investigation of the genetic backgrounds of target catches. Genetically based methods have a wide range of applications to twenty-first-century fishery management and germplasm resource protection [19]. Genetic approaches have provided a great deal of insight into stock structure and species identification, genetic diversity and divergence, as well as historical dynamics and local adaptation in marine species [20]. At present, next-generation sequencing (NGS) is continuously changing the landscape of genomics, making the discovery of molecular markers of the whole genome cost-effective [21]. Genotyping-by-sequencing (GBS) is a popular reduced-representation genome sequencing (RRGS) technique that allows the identification of high-density single-nucleotide polymorphisms (SNPs) to reveal genetic variations in non-model species at the genomic level [22], and its applications have covered various fields in genetic linkage mapping, stock identification, molecular conservation, population genetics, and evolutionary relationships in fisheries and aquaculture [23]. Particularly in research on the population genomics of marine species, the large number of polymorphisms enables the characterization of population genetic diversity and structure using a handful of samples [24].

Here, a novel GBS protocol was applied to detect genome-scale SNPs in *C. feriata* from four different geographical regions along China’s coasts. The main aims of this present study were as follows: (1) revealing the genetic diversity of *C. feriata* at the genomic level; (2) examining the genetic differentiation of *C. feriata* and its driving forces; and (3) characterizing population structures and spatial patterns of *C. feriata* across the southeast China Sea. Our results are desirable to fill gaps in knowledge regarding population genomics and the need for resource protection and also serve as meaningful references for the exploitation and utilization of *C. feriata* resources in China.

## 2. Materials and Methods

### 2.1. Sample Collection and DNA Extraction

**Figure 1:**
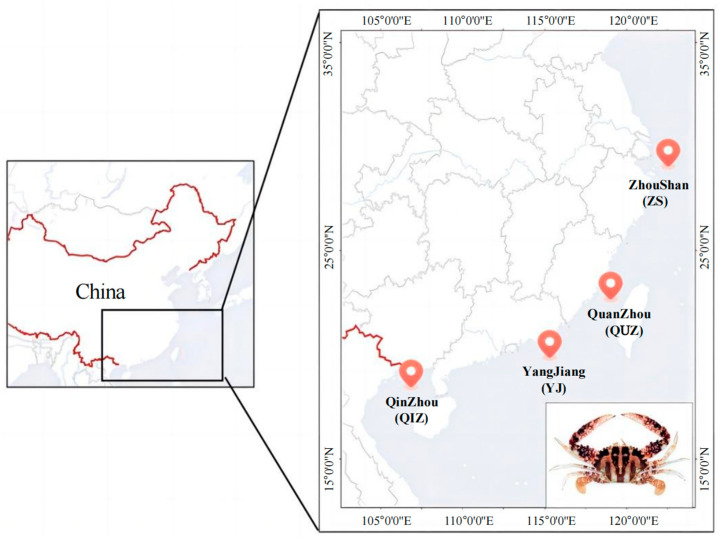
The sampling locations of *C. feriata*.

A total of 60 wild individuals of *C. feriata* were collected from north to south through the southeast coast of China in September 2023, with the sampling sties including Zhoushan, Zhejiang Province (ZS, n = 15); Quanzhou, Fujian Province (QUZ, n = 15); Yangjiang, Guangdong Province (YJ, n = 15); and Qinzhou, Guangxi Province (QIZ, n = 15), respectively (Figure 1). A bit of musculature was excised and soaked in 95% ethanol immediately and then preserved in an ultra-low temperature freezer (−80 °C) for the following experiments. Total genomic DNA was extracted using the standard phenol–chloroform method [25]. The integrity and concentration of DNA were detected by 1% agarose gel electrophoresis and the NanoDrop 8000 Spectrophotometer (Thermo Scientific, Waltham, MA, USA), respectively.

### 2.2. Library Preparation and Sequencing

The paired-end libraries with an insert size of 300 bp were constructed by Shanghai OE Biotech Co. Ltd. (Shanghai, China), according to the UGbS-Flex preparation protocol [26]. The high-throughput sequencing library construction procedure is briefly described as follows: (1) The genomic DNA was digested by 30 μL *Pst*I-HF (8 units) and *Msp*I (8 units) restriction enzymes (NEB, Ipswich, MA, USA) at 37 °C for 2 h. (2) The digested DNA fragments were examined by electrophoresis and then ligated to adaptors with unique barcodes using T4-DNA ligase (NEB, USA) in a total volume of 40 μL at 22 °C for 2 h. (3) DNA fragments with a length of 300–700 bp were recovered by incubating the samples with 0.7 volumes of Sera-Mag SpeedBeads (GE Healthcare Life Sciences, Issaquah, WA, USA) at room temperature for 5 min. (4) PCR amplification was conducted for each sample separately using an initial denaturation at 95 °C for 30 s, 16 cycles of denaturation at 95 °C for 30 s, primer annealing at 62 °C for 20 s, and fragment elongation at 68 °C for 15 s, followed by a final fragment elongation step at 68 °C for 5 min. (5) PCR products were checked on a 1.5% agarose gel and the DNA concentration (>5 ng/μL) of each GBS library was measured on the Qubit 2.0 Fluorometer using a Qubit™ dsDNA HS assay kit (Invitrogen, Waltham, MA, USA). (6) Moreover, 30 ng of each GBS library was pooled, and primers, dNTPs, and small DNA fragments were removed. Finally, the pooled GBS libraries (100 ng) were sequenced on the Illumina NovaSeq PE150 platform.

### 2.3. Quality Control and Read Mapping

Sequencing data generated by GBS-seq were checked with the FastQC program (http://www.bioinformatics.babraham.ac.uk/projects/fastqc; accessed on 16 May 2024), and then reads were split by a barcode using the module “process_radtags” within the Stacks pipeline v2.4 [27]. The obtained raw reads were purified by removing adaptors, cohesive ends, and low-quality reads (N base ≥ 5 bp, Phred quality score < 20, read length < 130 bp) by using fastp software v0.20.0 [28]. The *Portunus trituberculatus* (Portunidae, *Portunus*) genome was chosen as a reference [29] because no chromosome-level genome of *C. feriata* was available from the public database. The clean reads were mapped to the reference genome using BWA software v0.7.17 with the default settings [30].

### 2.4. Genomic SNPs Calling and Annotation

The Haplotypecaller module of GATK v4.1.3 was used to call out the genomic SNPs [31]. In order to obtain robust results in the subsequent analyses, the following exclusion criteria were applied for variants filtered by VCFtools v0.1.16 [32]: loci with sequencing depth < 4, MAF (SNPs with a minor allele frequency) < 0.01, and genotyping rate < 80%. Then, SnpEff v4.1g was adopted to annotate the filtered SNPs [33].

### 2.5. Population Genetics Analysis

For population genetic analysis, each genotype with markers was assembled head to tail and missing sites were replaced by “–”. Genetic diversity parameters containing nucleotide diversity (*π*), expected heterozygosity (*H*e), observed heterozygosity (*H*o), the effective number of alleles (*N*ea), and polymorphism information content (*PIC*) were calculated by VCFtools v0.1.16 [32]. The *π* value is estimated by the number of nucleotide substitutions between two populations, and heterozygosity is defined as the proportion of heterozygotes amongst all genotypes within a population. As a traditional manifestation of genetic variability, heterozygosity can be characterized as observed and predicted heterozygosity [34]. *N*ea is a common measure of genetic variability introduced by Kimura and Crow in 1964, and it refers to the reciprocal of the probability that two randomly chosen homologous genes in the population are identical by descent [35]. The level of polymorphism can be assessed by the *PIC* value as follows: very informative (>0.5), moderately informative (0.25~0.5), and poorly informative (<0.25) [36].

Wright’s F-statistics were first proposed by Wright in 1921 [37] and then extended and improved by several other scholars [38]. The F-statistics of each SNP locus were used to evaluate the genetic differentiation of different populations by Genepop v4.0 [39]. The pairwise fixation index (*F*st) and its corresponding *p* value were calculated by StAMPP v1.6.3 in R package [40]. Reynolds’ genetic distance (DR) between populations was estimated through *F*st, with the formula DR = −ln (1 − *F*st) [41].

A neighbor-joining (NJ) tree was constructed with Treebest v1.9.2 under the p-distance model, using bootstrapping with 1000 replicates [42]. The R package ggtree v1.16.6 was used for visualizing the phylogenetic tree. A principal component analysis (PCA) of the obtained SNPs was implemented in GCTA v1.26.0 to obtain the most influential feature vectors [43] after converting the files with PLINK v1.9 [44]. To determine the optimal value of population number (K) and population structure, the SNP genotyping information was analyzed via ADMIXTURE v1.3.0 [45].

## 3. Results

### 3.1. GBS-Seq Data Processing and Genotyping

GBS-seq of 60 *C. feriata* individuals generated a total of 95.06 Gb raw data, with an average sequencing depth of 10×. After sequence quality control, the number of high-quality clean reads per sample ranged from 6,478,730 (ZS09) to 11,373,324 (QUZ01), with an average number of clean reads of 9,203,798. Most genotype rates of clean reads were higher than 93%. The GC content, Q20 value, and Q30 value were 48.46%, 97.32%, and 93.07%, respectively, indicating the qualified data met the requirements of the subsequent analysis (Table 1).

**Figure 2:**
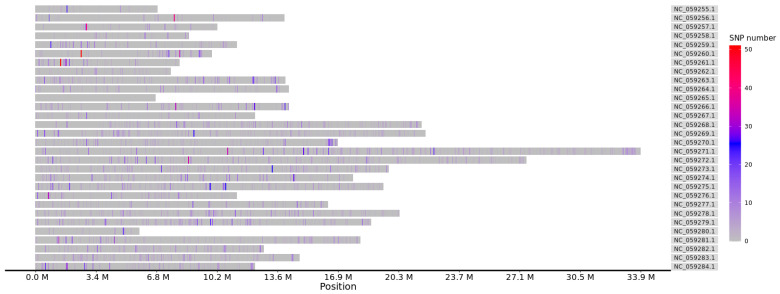
The distribution of SNPs on different chromosomes of *C. feriata*.

After sequence alignment and variant identification, a total of 976,425 pre-filtered SNPs were detected, and 18,815 SNPs were ultimately retained after screening. The distribution of genome-wide SNPs is presented in Figure 2, showing that variants were unevenly distributed on each chromosome (30 chromosomes in total). NC_059271.1, NC_059272.1, and NC_059279.1 were the top three chromosomes with the most SNPs per 1000 bp, whereas chromosome NC_059265.1 had the fewest SNPs per 1000 bp.

**Figure 3:**
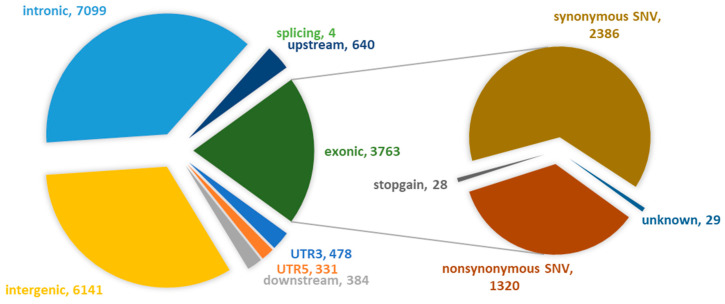
Annotation distribution of SNPs in the genome of *C. feriata*.

Most of the SNPs were located in the intronic region (7099) and intergenic region (6141). About 3763 variable loci were found in the exonic region, including synonymous SNPs (2386), nonsynonymous SNPs (1320), stop gain (28) and unknown (29), respectively (Figure 3).

### 3.2. Genetic Diversity

To determine the genetic variation among four *C. feriata* populations, the *π*, *H*e, *H*o, *N*ea, and *PIC* values of each SNP locus were separately estimated, and the statistical data are shown in Table 2. In the present study, the *π* values ranged from 0.2123 (QIZ) to 0.2226 (ZS), with an average of 0.2156. The overall *PIC* value was calculated to be 0.1742 (<0.25), indicting a low level of polymorphism associated with a locus. Among all sampling locations, *H*e varied from 0.2047 to 0.2146 with a mean of 0.2137, and *H*o from 0.2465 to 0.2484 with a mean of 0.2475. *H*o was higher than *H*e in all populations, suggesting that it appeared to exhibit an excess of heterozygosity. Moreover, we further assessed the *N*ea (1.3454~1.3609) of *C. feriata* populations and found that the values reduced as the latitude decreased, which could be also seen for *PIC* parameters.

### 3.3. Genetic Differentiation and Genetic Distance

The genetic differentiation level of four *C. feriata* populations was measured by F-statistics. Wright’s F coefficients involving the within-population inbreeding coefficient (*F*is), the overall inbreeding coefficient (*F*it), and the fixation index (*F*st) were determined to be −0.1648, −0.1585, and 0.0054, respectively, in all individuals at each SNP locus, estimating a 95% confidence interval with 1000 bootstraps. The pairwise *F*st value and the corresponding genetic distance were calculated to compare the genetic divergence between two populations (Table 3). The pairwise *F*st values (0.0012~0.0102, *p* < 0.01) were very low across different locations. An identical pattern was noticed from the results of genetic differentiation and genetic distance. The largest genetic differentiation and genetic distance were found between the ZS and QIZ populations, while the QUZ and YJ populations had the highest population genetic identity.

### 3.4. Population Genetic Structure

**Figure 4:**
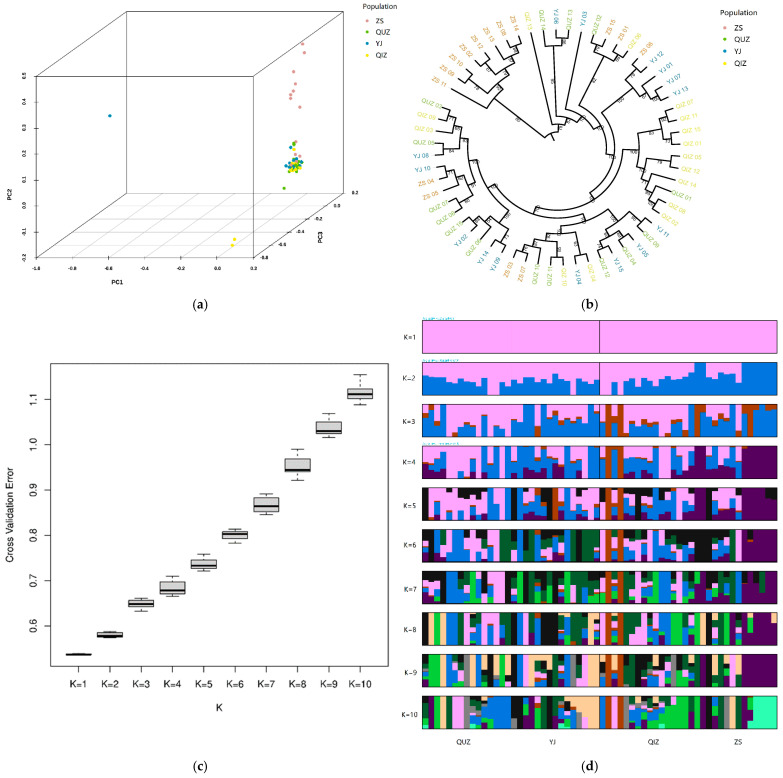
The population genetic structure of *C. feriata.* (**a**) PCA plot. (**b**) Phylogenetic tree. (**c**) Distribution of CV error values for different K values. (**d**) ADMIXTURE analysis.

PCA was conducted to analyze the population structure of *C. feriata* used in this study, and most of the measured variances could be explained by the first three principal components (PCs), with percentages of 31.8% (PC1), 29.5% (PC2) and 22.8% (PC3), respectively (Figure 4a). A three-dimensional scatter plot revealed that the majority of specimens from the four populations gathered together, but only a certain number of ZS individuals separated in one axis (PC2). The NJ phylogenetic tree found a consistent result, showing a nonsignificant clustering trend among *C. feriata* in different populations, except that eight individuals in the ZS population clustered to the outermost branch in the topological tree (Figure 4b).

To further understand the degree of admixture in *C. feriata* populations, the cross-validation (CV) error rate of the K value was set from 1 to 10, and the best K (the lowest peak) value was 1 by varying the number of presumed ancestral populations (Figure 4c). All the populations formed one cluster and originated from the same ancestor at K = 1. The admixture analysis showed that populations were highly mixed with an increase the K value, which provided strong support for the high genetic homogeneity of *C. feriata* (Figure 4d). Furthermore, the clustering relationships demonstrated that almost one-half of the ZS population were separated as a unique cluster when K values were in the range of 2 to 10, implying that these samples might have genetic divergence with others.

## 4. Discussion

Unraveling the genetic background of fishery species is an essential prerequisite and basis for effective fishery management and sustainable resource utilization [46]. Additionally, parents often have important influences on their offspring’s traits and fitness. Both the phenotypes and genotypes of offspring are considerably related to the genetic characteristics of their wild parents, caught from their natural surroundings [47]. The swimming crab *C. feriata* is not only an important economic crustacean in Chinese and overseas markets, but it has also been regarded as a valuable aquaculture target given its meat quality, taste, and size. Nowadays, artificial propagations have gradually been carried out in order to alleviate market supply–demand relationships [12,13,48]. Especially with the ongoing development of the global crab aquaculture industry, it is necessary to conduct further population genetic research on *C. feriata* from the point of view of fishery management and aquaculture practice.

Considering the limited capability of a single genetic marker to identify genetic information, in the presently reported study, we unveiled the genetic diversity and population structure of *C. feriata* along the southeastern coast of China based on SNPs across the whole genome and using GBS-seq for the first time. Genetic diversity is crucial for the adaptability and survivability of species, which is typically quantified by *H*e for its lower susceptibility to sample size than actual heterozygosity, *H*o [49]. Consistent with the findings of Ma et al. [17], all of the populations of *C. feriata* in this study exhibited relatively higher levels of genetic diversity compared to some other marine crustacean species, such as *Callinectes sapidus* (*H*e = 0.103~0.108) [50], *Oratosquilla oratoria* (*H*e = 0.0810~0.1165) [51], and *Homarus gammarus* (*H*e = 0.120~0.146) [52], manifesting abundant genetic information and rich genetic variation in *C. feriata* populations. In addition, the greater values of *H*o compared to those of *H*e in all loci suggested an excess of heterozygosity, which perhaps resulted from a higher probability of survival for heterozygous individuals. It was worth mentioning that the ZS population possessed the highest genetic diversity and variability. The Zhoushan Archipelago is located on the southern side of the Yangtze River estuary and serves as an important spawning, feeding, and nursery ground for many fishery species. Maybe the suitable habitat environment in the Zhoushan Archipelago sea area was the critical factor for maintaining the high genetic diversity of natural populations. Similar conclusions were also obtained in several species living in this sea area, for example, *Sepiella japonica* [53], *Scapharca subcrenata* [54], *Oplegnathus fasciatus* [55], and *Acanthogobius ommaturus* [56].

The extremely low values of *F*is and *F*it demonstrated a prevalence of less within-population inbreeding and random mating among different populations [57]. A weak genetic differentiation (*F*st < 0.05) was detected [58], which was probably caused by an extensive gene exchange during the pelagic egg and larvae phases of most crustaceans (a duration of 2~3 months). The long-distance larval dispersal by marine currents allowed gene flow across broad expanses of the oceans and potentially provided genetic cohesion within widespread marine carbs, just like *Scylla paramamosain* [59], *Portunus trituberculatus* [60], *Callinectes sapidus* [50], *Cancer magister* [61], and *Metacarcinus edwardsii* [62]. Genetic divergence between the ZS and QIZ populations was the greatest, which might be related to the long distance between these two geographical regions. In contrast, the closest genetic connectivity was that of the QUZ and YJ populations, indicating free interbreeding and gene flow between them. The results of genetic distance also showed the same results, which confirmed a certain correlation between genetic distance and geographic distance.

The population genetic structure of *C. feriata* revealed by PCA, ADMIXTURE, and phylogenetic analyses, in particular, exhibited no obvious genetic pattern in the four sampling localities. Notwithstanding, we still discovered a certain genetic heterogeneity in the ZS population from the results of PCA and the NJ tree. We speculated that the diluted surface water from the Yangtze River Estuary might have formed a geographic barrier of *C. feriata* and divided the ZS population into different subpopulations, which were influenced by specific geographical environments, ecological factors, and living habits. Mutations and genetic drift might be the main drivers in promoting the increase in intra-population genetic differences. Owing to the locational uniqueness of the Zhoushan Islands, we suggest that an independent fishery management policy for *C. feriata* should be established here, whereas the remaining populations of *C. feriata* distributed in the East China Sea and the South China Sea could be treated as an integrated fishery management unit.

## 5. Conclusions

The study of the genetic diversity and population structure between and within populations is essential for the effective management of genetic resources. In this study, we applied a population genomics strategy to investigate the genetic background of *C. feriata*. Generally, the results showed that *C. feriata* populations in the southeast coast of China maintained a relatively higher genetic diversity and displayed very weak genetic differentiation. Notably, *C. feriata* in Zhoushan fishing grounds exhibited extensive genetic variability and complexity, which might be associated with the specific habitat of archipelagic waters. The conclusions drawn from our study will shed light on the germplasm resource assessment and fishery management of *C. feriata* in the future.

## Figures and Tables

**Table 1:** Summary of sequencing data in four *C. feriata* populations.

	Index	Average Raw Reads	Average Clean Reads	Genotype Rate (%)	GC Content (%)	Q20 (%)	Q30 (%)
Population							
ZS		9,383,552	8,381,687	93.20	47.95	97.03	92.42
QUZ		11,541,551	9,760,331	93.80	48.54	97.43	93.29
YJ		11,501,662	9,660,050	94.00	48.69	97.41	93.25
QIZ		10,793,920	9,013,123	93.46	48.65	97.43	93.30
Total		10,805,171	9,203,798	93.62	48.46	97.32	93.07

**Table 2:** Genetic diversity parameters of *C. feriata* populations based on the whole-genome SNPs.

	Index	Nucleotide Diversity (*π*)	Expected Heterozygosity (*H*e)	Observed Heterozygosity (*H*o)	Effective Number of Alleles (*N*ea)	Polymorphism Information Content (*PIC*)
Population						
ZS		0.2226	0.2146	0.2482	1.3609	0.1738
QUZ		0.2144	0.2068	0.2465	1.3492	0.1672
YJ		0.2135	0.2060	0.2484	1.3473	0.1667
QIZ		0.2123	0.2047	0.2469	1.3454	0.1656
Average		0.2156	0.2137	0.2475	1.3556	0.1742

**Table 3:** The genetic differentiation (below the diagonal) and genetic distance (above the diagonal) of *C. feriata* populations inferred from Wright’s *F*st and Reynolds DR, respectively.

	ZS	QUZ	YJ	QIZ
ZS	—	0.0074	0.0082	0.0103
QUZ	0.0074 **	—	0.0012	0.0024
YJ	0.0082 **	0.0012 **	—	0.0042
QIZ	0.0102 **	0.0024 **	0.0042 **	—

The double asterisk (**) represents extremely significant difference (*p* < 0.01).

## Data Availability

The raw data supporting the conclusions of this article will be made available by the authors upon request.
